# Thyroid Hormone Response Element Half-Site Organization and Its Effect on Thyroid Hormone Mediated Transcription

**DOI:** 10.1371/journal.pone.0101155

**Published:** 2014-06-27

**Authors:** Martin A. Paquette, Ella Atlas, Mike G. Wade, Carole L. Yauk

**Affiliations:** 1 Environmental Health Science and Research Bureau, Healthy Environments and Consumer Safety Branch, Health Canada, Ottawa, Ontario, Canada; 2 Department of Biology, Carleton University, Ottawa, Ontario, Canada; Florida International University, United States of America

## Abstract

Thyroid hormone (TH) exerts its effects by binding to the thyroid hormone receptor (TR), which binds to TH response elements (TREs) to regulate target gene expression. We investigated the relative ability of liganded homodimers TR and retinoid X receptor (RXR), and the heterodimer TR/RXR, to regulate gene expression for the TRE half-site organizations: direct repeat 4 (DR4), inverted repeat 0 (IR0) and everted repeat 6 (ER6). Luciferase reporter assays using a DR4 TRE suggest that both the TR homodimer and TR/RXR heterodimer regulate luciferase expression in the presence of their respective ligands. However, in the presence of the IR0 TRE, transfection with TR/RXR and RXR alone increased luciferase activity and there was no effect of TR alone. The presence of 9-cis-retinoic acid was necessary for luciferase expression, whereas TH treatment alone was insufficient. For the ER6 TRE, transfection with TR/RXR, TR alone and RXR alone (in the presence of their respective ligands) all caused a significant increase in luciferase activity. When both ligands were present, transfection with both TR/RXR caused more activation. Finally, we investigated the efficacy of the TR-antagonist 1–850 in inhibiting transcription by TR or TR/RXR at DR4 and ER6 TREs. We found that 1–850 did not suppress luciferase activation in the presence of TR/RXR for the ER6 TRE, suggesting conformational changes of the ligand binding domain of the TR when bound to different TRE half-site organizations. Collectively, the findings indicate that there are fundamental differences between TRE configurations that affect nuclear receptor interactions with the response element and ability to bind ligands and antagonists.

## Introduction

Thyroid hormones (THs) regulate genes involved in many different functions, from metabolism to neuronal development. Disruption of TH signalling can lead to detrimental effects, especially during gestation where minor alterations in TH levels can cause long-term neurophysiological impairment to the child [1]. Iodine deficiency is the most common cause of hypothyroidism and affects almost one-third of the world's population [2]. TH levels can also be altered by exposure to environmental contaminants such as bisphenol A, polychlorinated biphenyls and polybrominated diphenyl ethers [3]–[6]. Understanding the molecular mechanics of TH action to control gene expression will facilitate the identification and mitigation of environmental factors that impair TH signalling.

Gene regulation by TH is achieved through activation of the TH receptor (TR). TR is bound to thyroid hormone response elements (TREs) in DNA in the presence or absence of TH. Interaction of triiodothyronine (T3) with TR causes conformational changes in TR and activation (or sometimes suppression) of target genes (see [7] for review). TREs are generally composed of two half-sites; the classic half-site is an AGGTCA hexamer [8], [9]. However, the TREs that have been identified and characterized thus far are often not composed of perfect classic half-sites, but instead tend to be degenerated. The orientation and spacing of the half-sites can also vary across TREs, although three types of TREs are the most well-known: i) direct repeat 4; ii) inverted repeat 0; and iii) everted repeat 6. Although most characterized TREs have been shown to positively regulate gene expression, negative TREs (nTREs) have also been observed [10]–[13]. This adds an additional layer of complexity in the identification and characterization of TREs.

The most frequently characterized TRE consists of a direct repeat of the half site with a 4bp spacer (DR4) between half sites. This can also be described as head to tail organization. A good example of a DR4 TRE is found in the promoter region of kruppel-like factor 9 (*Klf9*), and has been characterized in mouse, rats and humans [14] as AGGTGAagtgAGGTCA (mouse sequence). A second type of TRE is composed of an inverted repeat (or palindrome – Pal0) with no spacer between half sites in a head to head organization (IR0). This is the least well characterized TRE and very few IR0s have been identified. One of the first TREs to be characterized was the rat growth hormone 1 (*Gh1*) promoter TRE. This TRE is comprised of a combination of organizations including an IR0 TRE [15], [16]. A third type of TRE is an everted repeat with a 6bp spacer (ER6 – sometimes referred to as F2 or IP6), referred to as a tail to tail organization. The myelin basic protein (*Mbp*) promoter regions in the mouse and rat have ER6 TREs [17]; the mouse *Mbp* TRE sequence is GGACCTcggctgAGGACA.

The TR can operate as a homodimer to drive gene expression but it is also frequently heterodimerized with the retinoid X receptor (RXR) [18]. The ligand-binding domain (LBD) of TR primarily binds to T3, the most active form of TH, whereas the RXR LBD binds 9-cis-retinoic acid (9cRA) [19]–[21]. In the TR/RXR heterodimer, RXR was once considered to be non-permissive; i.e., 9cRA was not able to transactivate target gene expression [22]. In this model, RXR was thought of as a silent partner. Further investigation led to the discovery that RXR was not a silent partner and could, in fact, bind its ligand and affect transcription [23]. More importantly, it is now understood that RXR and its ligand participate in regulatory activities of the TR/RXR heterodimer [24].

In this study we investigate the relative ability of liganded TR, RXR and TR/RXR to drive gene expression in respect to TRE half-site organization – DR4, IR0 and ER6. We also investigate the efficacy of a TR-antagonist in relation to TRE half-site organization in the presence TR and TR/RXR. Our results reveal that there are important differences between the TRE organizations that relate to the presence of binding partners and their ability to drive gene expression. The results also suggest that TRE organization may affect the ability of TR-antagonists to inhibit transcription.

## Materials and Methods

### Plasmid Construction

Endonuclease restriction sites were added to each custom oligonucleotide (Eurofins MWG Operon, KY, USA) to allow for directional cloning into pGL4.10[luc2] vector (Promega, Madison, WI, USA). Oligonucleotides were annealed, followed by phenol chloroform cleaning. Oligonucleotides and plasmids were digested by XhoI and HindIII (New England Biolabs, ON, Canada) for 1h and ligated overnight using T4 Ligase (New England Biolabs). Plasmids were then transformed into Mach1-T1R chemically competent *E. coli* bacteria (Invitrogen, Burlington, ON, Canada) as per the manufacturer's recommendations, and spread onto ampicillin plates to be grown overnight. A number of colonies were selected to be grown in Luria-Bertani broth + ampicillin, followed by plasmid isolation using QIAprepr Spin Miniprep kit (Qiagen, Missisauga, ON, Canada). Plasmids containing DR4, IR0 or ER6 TREs were verified by sequencing (see Table 1 for oligonucleotide sequence).

**Table 1:** Half-site organization for TRE constructs.

TRE	Sequence (5′ to 3′)
DR4	CTCGAG **AGGTCA**CTTC**AGGTCA**TCTACGTAACTGATGT**AGGTCA**CTTC**AGGTCA** AAGCTT
IR0	CTCGAG **AGGTCATGACCT**TCTACGTAACTGATGT**AGGTCATGACCT** AAGCTT
ER6	CTCGAG **TGACCT**CGGCTG**AGGTCA**TCTACGTAACTGATGT**TGACCT**CGGCTG**AGGTCA** AAGCTT

Half-sites are in bold and the underlined sequences are the endonuclease sites (Xho I on left and Hind III on right) showing where the oligonucleotide was inserted into the plasmid.

The DR4 half-site spacer “CTTC” was chosen because this spacer is present in the human thyroid hormone responsive SPOT14 (*Thrsp*) TRE, which is one of the most well characterized TRE. The ER6 half-site spacer “CGGCTG” was chosen because it appears in the mouse *Mbp* TRE and is almost completely identical (5 out of the 6 bp are conserved) to the rat *Mbp* TRE spacer “CGGCCG”. Each plasmid contains two TREs separated by the same 16 bp spacer. The 16 bp space was randomly generated although it was verified not to contain GG dinucleotides since they appear to play an important role in TRE half-site functionality.

### Cell Culture, Transfection and Luciferase Reporter Assays

COS-7 cells were seeded in 12-well plates at a density of 4×10^4^ cells per well. Cells were grown in Dulbecco's Modified Eagle Medium containing 5% charcoal stripped fetal bovine serum and 0.5 mg/ml penicillin-streptomycin and were maintained at 37°C with 5% CO2. Twenty-four hours after seeding, cells were transfected using FUGENE HD (Promega) with 100 ng of the reporter plasmid of interest (see Table 1), and co-transfected with 10 ng pRL-CMV(Promega) and 50 ng TRα (SC307938, Origene, MD, USA) with and without 50 ng RXRα (MC216284, Origene). Twenty-four hours post-transfection cells were treated with T3 (5 nM) (3,3′,5-triiodo-L-thyronine, Sigma Chemical, Oakville, ON, Canada) and/or 9cRA (1 µM) (Santa Cruz Biotechnology Inc., CA, USA). Twenty-four hours after hormone treatment cells were harvested and firefly luciferase activity was determined using a Veritas luminometer with Dual-Luciferase Reporter Assay System (Promega). Firefly luciferase activity was normalized to renilla luciferase activity. Experiments were run in triplicates and repeated at least three times independently (i.e., on separate days).

For TR antagonist 1–850 (EMD Milipore, MA, USA) exposures, cells were transfected (as described above) 5 h after seeding. Twenty-four hours after transfection, cells were treated with 10 µM 1–850 in DMSO. Twenty-four hours after 1–850 exposure, cells were treated with T3 (5 nM), and harvested 24 h later. Previous work has shown that there were not significant differences between a 5 h cell adhesion and 24 h cell adhesion (as previously described). The 5 h cell adhesion was used for this set of experiments due to timing issues.

All of the experiments described above were run in triplicates and repeated at least three times. Fold-changes were calculated by taking the average of triplicates to calculate ratios. These ratios were then log transformed to calculate an average and then reverse log-transformed. Statistical significance was determined by a two tailed, paired Student's t-test or one way ANOVA followed by Tukey's HSD test (as indicated in figure legends).

## Results

### TH and 9cRA Response for Three TRE Configurations

Three different TRE configurations – DR4, IR0 and ER6 were investigated for their potential to drive gene expression in the presence or absence of TR and/or RXR when treated with T3 and/or 9cRA. Oligonucleotides were designed to contain two TREs separated by an identical 16 bp spacer (see Table 1) and were cloned into the same two restriction sites to allow for directional cloning into pGL4.10. COS-7 cells were selected for this experiment due to their ease of use and high transfection efficiency. Additionally, the TRE expression plasmids do not respond to their respective ligands in the absence of TR and/or RXR co-transfection, indicating very low levels of TR and RXR; these findings confirm that COS-7 cells are a good choice for evaluating TRE activation in response to specific nuclear receptor binding.

**Figure 1:**
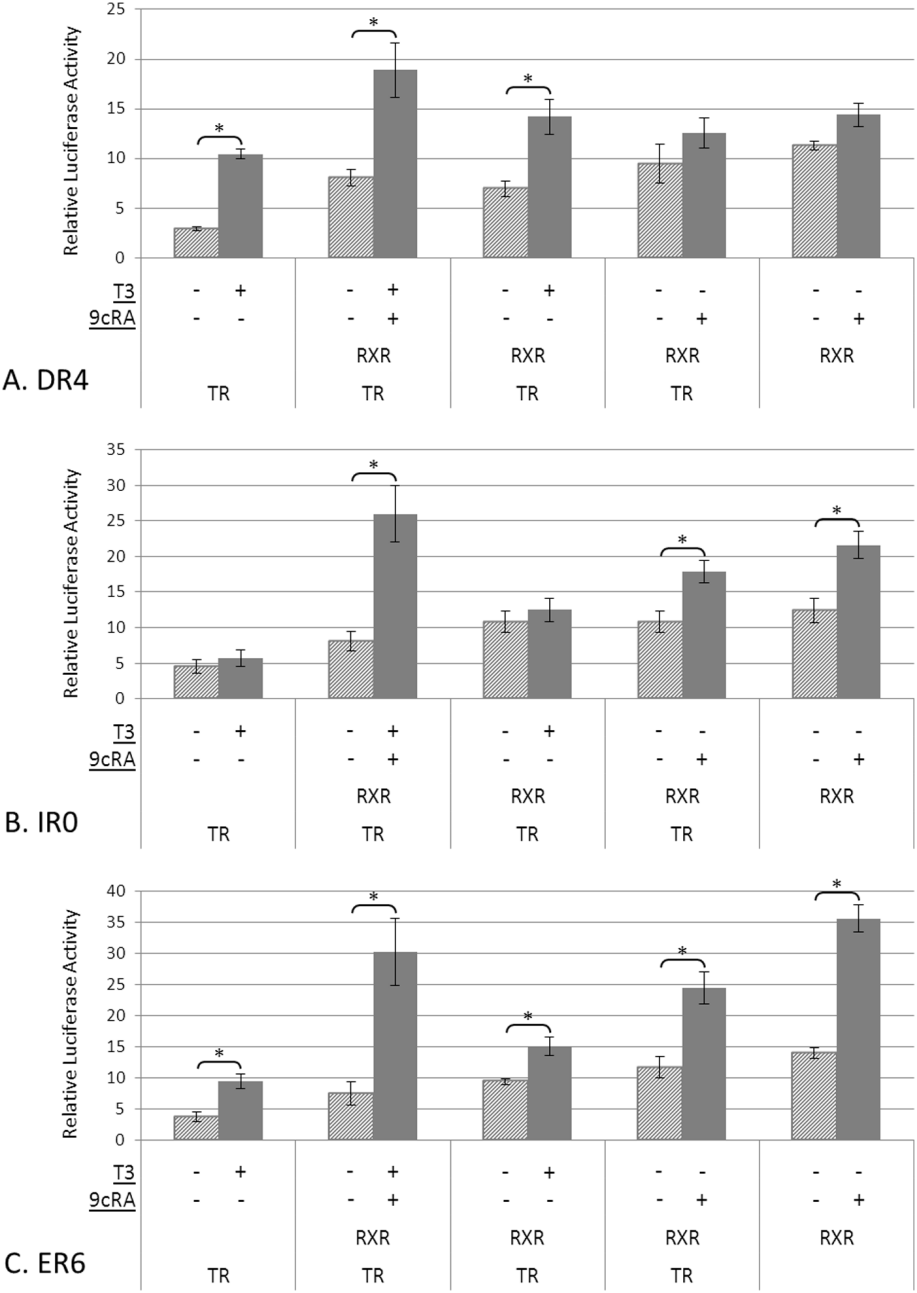
Transcriptional activity determined by luciferase reporter assay for DR4 (A), IR0 (B) and ER6 (C). COS-7 cells were co-transfected with a TRα and/or RXRα expression vectors. Cells were treated with 5 nM T3 and/or 1 µM 9cRA alongside untreated controls. Firefly luciferase expression was normalized to renilla luciferase expression. Experiments were run in triplicate and repeated at least 3 times. Data for each condition are shown separately since each experiment is paired and was run independently. Data are presented as means ± standard error of the mean. Asterisks (*) denote a significant difference, p≤0.05, determined by a two-tailed, paired Student's t-test.

When evaluating the DR4 plasmid, co-transfection with TR resulted in a significant 3.6-fold increase in luciferase activity in the presence of T3 (Figure 1A). Cells co-transfected with RXR in addition to TR exhibited a significant 2.3-fold and 2.0-fold increase in luciferase activity in the presence of T3+9cRA or T3 alone, respectively, when compared to vehicle control. When cells were transfected with TR+RXR or RXR alone, no significant changes to luciferase activity were observed when treated with 9cRA.

The organization of the IR0 construct is shown in Table 1, and the experimental results are summarized in Figure 1B. When co-transfected with TR only or TR+RXR, T3 treatment did not induce luciferase activity. However, when co-transfected with TR+RXR and treated with T3+9cRA, a significant 3.2-fold increase in activity was observed. A 1.7-fold increase was observed for cells treated with 9cRA alone when compared to baseline. When co-transfected with RXR only and treated with 9cRA, a significant 1.7 fold increase in luciferase activity was noted.

Results for the ER6 are shown in Figure 1C. When co-transfected with TR only and treated with T3 we observed a significant 2.5-fold increase in luciferase activity over non-T3 treated cells. When co-transfected with TR+RXR we observed a significant increase of 4.0-fold in the presence of T3+9cRA, 1.6-fold in the presence of T3, and 2.1-fold in the presence of 9cRA. In addition, when co-transfected with RXR alone and treated with 9cRA, a significant 2.5-fold increase in luciferase activity was detected.

### Effects of the TR antagonist 1-850 on TH-driven expression in the DR4 and ER6 plasmids

The chemical 1–850 has been shown to act as a TR antagonist under certain conditions [25]. Here, we examined the effect of 1–850 on two different TRE organizations in the presence of TR alone, or with TR+RXR. The IR0 TRE organization was not included in the analysis since we previously found that it was not responsive to T3 treatment alone.

**Figure 2:**
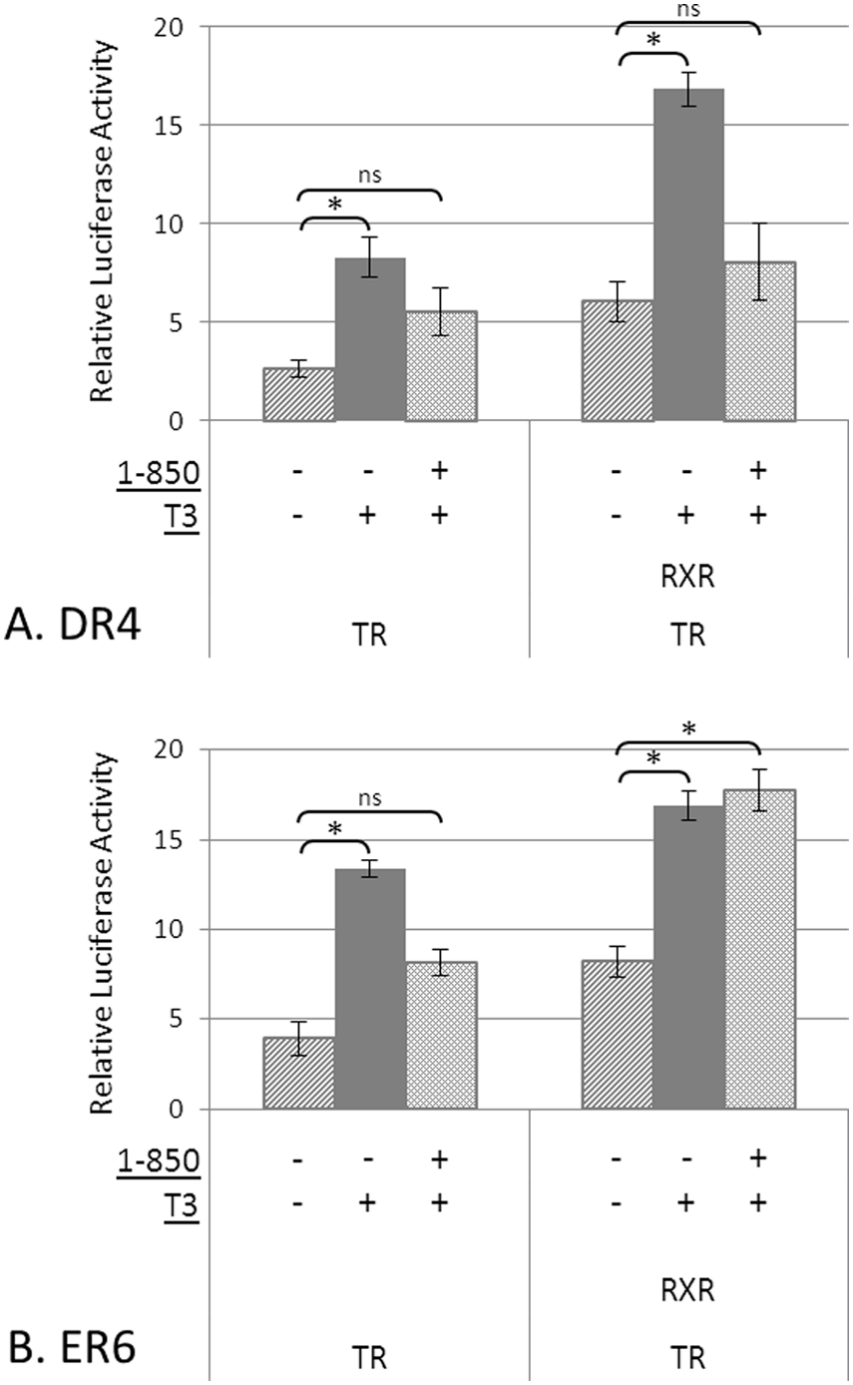
Antagonistic effects of 1–850 determined by luciferase reporter assay for DR4 (A) and ER6 (B). COS-7 cells were co-transfected with a TRα or TRα+RXRα expression vectors. Cells were treated with or without 5 nM T3, or with T3 in the presence of 10 µM 1–850. Firefly luciferase expression was normalized to renilla luciferase expression. Experiments were run in triplicate and repeated at least 3 times. Data are presented as means ± standard error of the mean. Asterisks (*) denote a significant difference, p≤0.05, determined by one way ANOVA followed by Tukey's HSD test; (ns) denote no significant difference, p≥0.05.

We observed a significant 3.2-fold increase in luciferase expression in DR4 plasmids in the presence of T3 (Figure 2A). However, in the presence of 1–850 (10 µM), this increase was inhibited by 48% compared to the T3 treatment alone. When co-transfected with TR+RXR, a significant 2.8-fold increase in luciferase expression was observed in the presence of T3, whereas in the presence of 1–850 a 1.3 fold increase was observed, which translates to a significant 75% inhibition when compared to T3 treatment alone. 1–850 alone did not show significant changes in luciferase activity in the presence of TR or TR+RXR (Figure S1A).

Experiments were repeated with the ER6 plasmid (Figure 2B). When co-transfected with TR, T3 induced a significant 3.4-fold increase in luciferase activity, whereas, with the addition of 1–850 a 1.6 fold increase was observed. Thus, 1–850 treatment caused a significant 48% reduction in luciferase activity compared to T3 treatment alone. Co-transfection of ER6 with TR+RXR and treatment with T3 induced a significant 2.1 fold increase in luciferase activity. However, there was no inhibitory effect of 1–850 on luciferase activity for ER6 when co-transfected with TR+RXR in the presence of T3 (i.e., fold increase remained consistent at a 2.1-fold increase). 1–850 alone did not show significant changes in luciferase activity in the presence of TR or TR+RXR (Figure S1B).

## Discussion

We investigated variables involved in TR-regulated gene expression for three established TRE sequence motifs. It is known that TRs can operate as monomers or in a complex as hetero- or homodimers to regulate the expression of TH-responsive genes. How sequence motif may influence the type and/or function of the nuclear receptor dimer that will form to control transcription has not been explored in detail. Thus, we examined whether TR and/or RXR, when treated with their respective ligands, were required for transcriptional activation when bound to the DR4, IR0 and ER6 TREs. Collectively, the results suggest that the three TRE organizations behave quite differently, that they do not all bind the same homo- or heterodimers, and that there may be both inhibitory and additive effects of co-activators and their ligands.

Using the DR4 TRE construct, TR activated gene expression in the presence of T3 based on luciferase activity (Figure 1A). When co-transfected with RXR and treated with T3+9cRA, or T3 alone, significant induction of transcription also occurred although fold-changes were significantly reduced (p = 0.004) when compared to TR alone. This finding is consistent with previous studies that have determined that RXR in the heterodimer TR/RXR can inhibit T3-mediated transactivation [23], [24]; this was shown using chloramphenicol acetyltransferase (CAT) reporter assays with a Gal4-TR chimera/Gal4 reporter model, as well as with a DR4 plasmid. The Li et al. 2004 study also found that inhibition by RXR was further enhanced in the presence of 9cRA, although this was not consistent with our experiments. We found that when using the DR4 construct, 9cRA was unable to increase luciferase activity in the presence of TR/RXR or RXR alone. Our findings suggest that when acting on a DR4 TRE, TR and T3 are more effective in driving gene expression on their own than in the presence of RXR and 9cRA.

In the presence of the IR0 TRE, TR and T3 were not able to activate gene expression (Figure 1B). Even when co-transfected with RXR, T3 alone did not significantly induce luciferase activity. However, RXR alone in the presence of 9cRA caused a significant increase in luciferase activity, suggesting that this type of half-site may act as a RXR response element (RXRE). This is supported by the fact that when co-transfected with TR and RXR, 9cRA alone caused a significant increase in luciferase activity. In the presence of 9cRA and T3, a marginal increase in gene expression was observed when compared to TR+RXR in the presence of 9cRA only (p = 0.14) or compared to RXR in the presence of 9cRA (p = 0.11). P-values are only approaching significance due to the variability of some of the experiments. Our results suggest that the IR0 response element allows binding of the RXR to activate gene expression, and provides some evidence that the binding of the TR/RXR may occur and operate on gene expression when both respective ligands are present. Additional work would be needed to validate these findings and confirm that TR interacts with this IR0. The work strongly suggsests that 9cRA is critical for gene expression activation by a TR/RXR heterodimer when bound to the IR0 TRE. The IR0 remains one of the least understood response element. In previous studies, the IR0 response element was found to allow binding of: i) retinoic acid receptor/RXR (RAR/RXR) heterodimer [26]; ii) farnesoid X receptor/pregnane X receptor (FXR/PXR) heterodimer [27]; and iii) vitamin D receptor/RXR (VDR/RXR) heterodimer [27]. Clearly further research on the IR0 response element is required to determine how specificity of nuclear receptor binding is achieved. Flanking sequence and the specific expression co-activator and co-repressors may play an important role in determining this specificity.

In the presence of the ER6 TRE, TR and T3 treatment caused a significant increase in luciferase activity (Figure 1C). Co-transfection with RXR and treatment with T3 and 9cRA appeared to cause a greater effect on transcription, although this was not statistically significant when compared to the induction caused by TR+T3 (possibly due to large standard errors). This result is in contrast to the DR4 TRE, for which a repression of TH-induced luciferase activity was noted in the presence of RXR. For ER6, both T3 and 9cRA on their own were able to induce gene expression in the presence of TR/RXR. Fold changes for these experiments were significantly lower than when T3 and 9cRA were administered together (p = 0.02 for both comparisons). In addition, cells transfected with RXR and treated with 9cRA also showed increase gene expression. The findings suggest TR homodimers, RXR homodimers and TR/RXR heterodimers are all able to effectively bind to the ER6 TRE and activate the target gene. The results also suggest that T3 and 9cRA may have an additive effect on transcriptional activation in the presence of TR/RXR heterodimer. In other words, the heterodimer can be activated by T3 or 9cRA although when both ligands are present significantly more activation occurs. A previous study on the prolactin (*Prl*) promoter region revealed similar findings [28]. These authors used a CAT reporter assay in Hela cells that was co-transfected with TR and RXR. CAT activity was increased in the presence of T3 or 9cRA. Interestingly, in the presence of both ligands an even higher transcriptional activation was observed. The active TRE in the promoter region of *Prl* has not been completely characterized and the half-site organization remains unconfirmed; our findings suggest that the TRE in question could be an ER6 since none of the other TRE organizations respond in this specific manner to these ligands.

Although we did not specifically test for protein-protein interactions, we assume that the transfection with TR or RXR proteins causes the formation of homodimers in the *in vitro* experiments, as well as different protein organisations, including monomers. The same can be said about TR/RXR. We assume that a large portion of the proteins form TR/RXR heterodimers (as often observed in *in vitro* experiments), although there is very likely the presence of TR or RXR monomers, dimers and multimers.

An overarching goal in our laboratory is to apply these TRE constructs to identify potential chemical TR agonists and antagonists. Thus, we explored the ability of a proposed TR antagonist (1–850) to inhibit TH-induced gene expression across the two TREs that responded to T3 (DR4 and ER6), to determine if chemical effects may also be specific to TRE sequence context. Specifically, we examined the effects of 1–850 on TR and TR/RXR promotion of gene expression when bound to a DR4 or ER6 TRE. The chemical 1–850 was previously identified through a virtual screening tool that searched for chemicals that could potentially bind the TR LBD [25]. When tested using a CAT reporter assay in Hela cells, 1–850 was found to inhibit TR transactivation by 80% at 5 µM concentration using a TRα expression plasmid and a reporter plasmid containing an IR0 TRE [25]. We note that we were unable to directly compare our findings with the results of Schapira et al. (2003) because our IR0 TRE plasmid when bound by TR homodimer or TR/RXR heterodimer was not significantly induced by T3.

In our experiments on COS-7 cells, 10 µM of 1–850 was used to inhibit TR activity. Much higher levels could not be used due to cellular toxicity. Viability tests using 10 µM of 1–850 did not significantly affect cellular growth (data not shown). Both DR4 and ER6 TREs that were co-transfected with a TRα expression plasmid in the presence of T3 exhibited comparable inhibition of T3 mediated transactivation in the presence of 1–850. However, inhibition caused by 1–850 in the presence of TR/RXR in DR4 plasmids was stronger than inhibition in the presence of TR only. This suggests that the TR/RXR heterodimer on DR4 may be more vulnerable to the inhibitory effects of 1–850, which may be due to differences in the structure of the LBD caused by heterodimerization. Surprisingly, inhibition by 1–850 in the presence of TR/RXR in the ER6 plasmid was not observed. This suggests that TR/RXR heterodimerization when associated with ER6 causes modifications to the LBD that permit T3 binding but inhibit 1–850 binding. Similar to other receptors, the TR LBD contains the specific region involved in dimerization [29]. The TR/RXR heterodimer binds to TREs that are composed of half-sites in various orientations with different sized spacers; therefore, it is reasonable to speculate that due to dimer configurations resulting from TRE organization, the LBD of the TR could have slightly different conformational or chemical properties that would cause a change in affinity to TH or TH-mimicking compounds. The overall results support the notion that TRE motifs govern the type and function of complexes formed on DNA to promote TH-driven gene expression.

Understanding TR-TRE mediated gene expression has remained a challenging task. This is in part due to the degenerate nature of these response elements. Our data demonstrate that the organization of the sequence motif is critical to TH activation of gene expression. The sequence motif adds a further layer of complexity to how the expression of a specific gene may be modified by changes in TH (or 9cRA) levels. We show that TRE configuration is likely to account for some of the differences observed between genes in the way that they respond to TH perturbations or exposures to chemicals that interact with TR. The effects of nucleotide substitution in the TRE half-site, alterations to flanking sequence and changes in half-site spacer composition would be interesting avenues to explore for future work in this area. These experiments are necessary to build stronger models for the different TREs to determine more precisely the genes and functions controlled by TH. Previous studies investigating the effects of various polychlorinated biphenyl (PCB) congeners on TH action have found that known positively TH-regulated genes such as RC3/neurogranin (*Nrgn*), *Thrsp*, and deiodinase 1(*Dio1*) respond differently in the presence of specific TH disruptors [30], [31]. Our findings in combination with those above suggest that in addition to 1–850, various PCB congeners may selectively inhibit TR/RXR heterodimers due to LBD differences that are caused by variations of TR-RXR interactions, which are specific to TRE half-site organization.

In conclusion, we have characterized the transcriptional responses of three different types of TREs to TH, 9cRA and a TR antagonist. We provide evidence that supports the hypothesis that RXR may be acting as a repressor when heterodimerized to TR bound to a DR4 TRE. We show that under the given conditions the IR0 TRE cannot be induced by T3 alone in the presence of TR or TR/RXR. We provide evidence of additive effects of T3 and 9cRA in the presence of TR/RXR for the ER6 TRE. Finally, the data suggest that the TR-antagonist 1–850 selectively inhibits TR/RXR heterodimers when bound to DR4 TREs but not ER6 TREs. Our findings are of critical importance for the development of *in vitro* chemical screening tools for TR-agonists or antagonists. These assays must take TRE organization into consideration. Cumulatively, these findings indicate that there are fundamental differences in TRE half-site organization that affect nuclear receptor interactions with response elements and ability to specifically bind their ligand and ligand mimics.

## Supporting Information

Figure S1 **Effects of 1–850 and T3 determined by luciferase reporter assay for DR4 (A) and ER6 (B).** COS-7 cells were co-transfected with a TRα or TRα+RXRα expression vectors. Cells were treated with or without 5 nM T3 or 10 µM 1–850. Firefly luciferase expression was normalized to renilla luciferase expression. Experiments were run in triplicate. Data are presented as means ± standard error of the mean. Asterisks (*) denote a significant difference, p≤0.05, determined by one way ANOVA followed by Tukey's HSD test; (ns) denote no significant difference, p≥0.05.
(PPTX)
