# Correction: Thyroid Hormone Response Element Half-Site Organization and Its Effect on Thyroid Hormone Mediated Transcription

**DOI:** 10.1371/journal.pone.0109226

**Published:** 2014-09-19

**Authors:** The PLOS ONE Staff

There are errors in Figure S1 of the published article. The correct File S1 can be viewed here.

## Supporting Information

Figure S1 **Effects of 1–850 and T3 determined by luciferase reporter assay for DR4 (A) and ER6 (B).** COS-7 cells were co-transfected with a TRα or TRα+RXRα expression vectors. Cells were treated with or without 5 nM T3 or 10 µM 1–850. Firefly luciferase expression was normalized to renilla luciferase expression. Experiments were run in triplicate. Data are presented as means ± standard error of the mean. Asterisks (*) denote a significant difference, p≤0.05, determined by one way ANOVA followed by Tukey's HSD test; (ns) denote no significant difference, p≥0.05.
(TIF)
